# Effectiveness comparisons of acupuncture for premature ejaculation: Protocol for a network meta-analysis

**DOI:** 10.1097/MD.0000000000014147

**Published:** 2019-02-01

**Authors:** Hengheng Dai, Haisong Li, Jisheng Wang, Binghao Bao, Yubing Yan, Bin Wang, Song Sun

**Affiliations:** a Graduate School of Beijing University of Chinese Medicine; b Department of Andrology, Dongzhimen Hospital, Beijing University of Chinese Medicine; c Chinese Medicine Hospital, Changping District, Beijing, China.

**Keywords:** acupuncture, meta-analysis, network

## Abstract

**Background:**

As one of the common male sexual dysfunction diseases, the treatment effect of premature ejaculation is often difficult to guarantee. In China, there are many randomized clinical trials that confirm that acupuncture has a good therapeutic effect on premature ejaculation. The aim of this study was to compare the efficacy and safety of acupuncture for premature ejaculation from intravaginal ejaculation latency (IELT), premature ejaculation diagnostic tool (PEDT), Arab premature ejaculation index (AIPE), and premature ejaculation index (IPE).

**Methods:**

We will use PubMed, EMBASE, Nursing and Related Health Literature Cumulative Index, Joint and Supplemental Drug Database, Cochrane Center Controlled Trials Registry (CENTRAL), China Biomedical Literature Database (CBM) and China Knowledge Infrastructure (CNKI), The Chinese Science and Technology Periodical Database (VIP), and Wanfang Database conduct systematic searches until October 31, 2018. At the same time, manually search for gray documents, including unpublished meeting articles. The primary outcome included intravaginal ejaculation latency (IELT). Secondary outcomes were premature ejaculation diagnostic tools (PEDT), Arab premature ejaculation index (AIPE), and premature ejaculation index (IPE). The quality and evidence that the risk of inclusion of the BiB tool will be assessed will be assessed on a scale.

**Results:**

This study will generate a comprehensive review of current evidence of acupuncture for premature ejaculation.

**Conclusion:**

The study will provide updated evidence to evaluate the efficacy and safety of acupuncture for premature ejaculation.

**Ethics and Communication:**

Because this research is based on a meta-analysis of published research, ethical recognition and patient consent are unnecessary.

**Agreement registration number:**

PROSPERO CRD42018111661.

## 1 Introduction

Premature ejaculation (PE) is the most common form of male sexual dysfunction, and published objective data is not sufficient to define evidence-based medicine for secondary PE.^[1]^ But nothing more than the following: short ejaculation latency, poor ejaculation control, and consequent low mood, can be divided into primary and secondary, recently, also proposed natural variability PE and premature ejaculation dysfunction 2 kinds of PE,^[2,3]^ but their specific role has not been clarified. Because of the specificity of the disease, there is currently no systematic assessment of primary PE and secondary PE, but most studies believe that the prevalence is between 20% and 30%.^[4,5]^ The prevalence of PE has nothing to do with age,^[6]^ more common in black, Spanish men, and men in Islamic backgrounds,^[7,8]^ and the prevalence of PE in men with lower education levels may be higher.^[9]^ At present, the cause of premature ejaculation is still unclear. The mainstream view is that it may be related to serotonin neurotransmitter disorder,^[10]^ excessive penile head sensitivity,^[11]^ genetic variation,^[12]^ prostatitis,^[13]^ thyroid disease, and emotional factors.^[14,15]^ PE can have a great psychological impact on patients, leading to mental distress, anxiety, and depression, which in turn has a major impact on the quality of life of patients.

The treatment of premature ejaculation should emphasize the implementation of personalized comprehensive treatment according to the classification diagnosis of patients’ diseases and different causes to improve the satisfaction of patients and partners. At present, the treatment of premature ejaculation includes medical treatment, behavioral psychotherapy, surgical treatment, and Chinese medicine treatment. Surgical treatment is still in the exploratory stage. Oral 5-HT receptor reuptake inhibitors (SSRIs) for the treatment of premature ejaculation are widely used in clinical applications, and can delay ejaculation to some extent,^[16]^ but have certain side effects, including nausea and diarrhea, headache, dizziness, decreased libido, no ejaculation, and erectile dysfunction.^[17,18]^ At the same time, the vast majority of psychotherapy outcomes are uncontrolled, single-blind, and few studies that meet the requirements of evidence-based medicine,^[19]^ the efficacy of behavioral therapy is difficult to maintain for a long time.^[20,21]^

In recent years, the application of acupuncture therapy has become more and more extensive, and clinically satisfactory treatment results have been obtained,^[22]^ but most of them are limited to clinical efficacy observation, based on the personal experience or preference of clinicians, lack of evidence-based exploration. This study used the method of mesh meta-analysis to directly or indirectly compare the efficacy of various acupuncture therapies and other therapies, and to sort the therapeutic effects, in order to provide evidence-based basis for the clinical decision-making of PE.

## 2 Methods

This is a systematic review and ethical approval was not necessary.

### 2.1 Study registration

Study protocol has been registered on PROSPERO CRD42018111661. (http://www.crd.york.ac.uk/PROSPERO/display_record.php?ID=CRD42018111661).

### 2.2 Eligibility criteria

#### 2.2.1 Type of study

All randomized controlled trials that investigate the effectiveness of ACU treatments for PE will be included.

#### 2.2.2 Participants

Patients must be over 18, any gender, disease stage, or severity.

#### 2.2.3 Interventions

The 2 groups of interventions are respectively the following treatment options: acupuncture combined with drug therapy or single acupuncture therapy (needle, moxibustion, cans, bee needles, fire needles, etc.), or comprehensive acupuncture therapy (needle, moxibustion, cans, bee needles, fire needles). Two or more acupuncture treatments; the use of materials, treatment sites, treatment time, and treatment are not limited, ACU treatment and drug treatment combined with the need to use the same drug treatment in the intervention group and the comparison group.

#### 2.2.4 Inclusion and exclusion criteria

##### 2.2.4.1 Inclusion criteria

Study types were randomized or quasi-randomized controlled trials, whether or not blinded. The document language is limited to Chinese and English. The 2 groups of interventions are any of the following treatment options: or single acupuncture therapy (needle, moxibustion, cans, bee needles, fire needles, etc.), or comprehensive acupuncture therapy (needle, moxibustion, cans, bee needles, fire needles, etc.). Therapy, the use of materials, treatment sites, treatment time, and treatment are not limited. Outcome indicators contain total efficiency.

##### 2.2.4.2 Exclusion criteria

Nonrandomized controlled clinical studies literature. Animal experiments, reviews, conference papers, etc will be excluded. The treatment plan involves other treatments such as traction, massage, and Chinese medicine. The results of the study are unclear and the research data is not detailed.

#### 2.2.5 Outcomes

The primary outcome measure is the intravaginal ejaculation latency (IELT), which is the time from when the penis is inserted into the vagina until the beginning of ejaculation. Secondary outcome measures were the Premature Effusion Diagnostic Tool (PEDT), the Arab Premature Eradication Index (AIPE), and the Premature Effusion Index (IPE). Each item of PEDT ranges from 0 (no problem) to 4 (very serious problem). The higher the score, the more severe the symptoms; AIPE and IPE are the opposite.

#### 2.2.6 Data source

Search online databases such as PubMed, Embase, Cochrane Library, China Biomedical Literature (CBM), China Knowledge Network (CNKI), VIP (VIP), Wanfang, etc., and collect acupuncture treatment of PE-related RCTs from October 2018. At the same time we will manually search conference proceedings, and the World Health Organization International Clinical Trial Registration Platform (ICTRP), ClinicalTrials.gov and the China Clinical Trial Registry. Search strategies will be performed as outlined in the Cochrane Handbook for Systematic Reviews of Interventions Version 5.1.0.

#### 2.2.7 Study selection

Data screening and extraction will be performed using EntNeX X6, EPDATA 3.1 and Excel 2013. Two researchers independently searched the above databases, read the topics and abstracts, removed the documents that did not meet the inclusion criteria, read the remaining documents in full, and determined the final eligible studies in strict accordance with the inclusion and exclusion criteria, such as 2 researchers. If there is a dispute between them, the decision will be discussed with the third researcher.

The extracted information includes: first author, publication year, sample size, total effective rate, randomized study and blinded information, return visits during study, shedding, and adverse reactions.

#### 2.2.8 Risk of bias

The literature quality assessment will be assessed using the Cochrane website's Risk of Bias (ROB) assessment tool.^[23]^ Evaluation criteria included random sequence generation (select bias), allocation concealment (select bias), results assessment blind detection (detection bias), incomplete outcome data (loss bias), selective reporting (report bias), and other biases. Two evaluators independently screened the literature according to the predetermined inclusion criteria and resolved the differences through discussion or a third researcher's assistance. If the data is missing, contact the author of the literature to obtain relevant information. According to the special data extraction form of this subject, one researcher extracts and enters the data, the other checks, uses the discussion to resolve the differences, and the lack of information is supplemented by the author of the literature communication.

#### 2.2.9 Statistical analysis

Statistical analysis was performed using the Bayesian model-based ADDIS software.^[24,25]^ Direct comparisons were performed using the Q test and I2 for heterogeneity testing. The odds ratio (OR) is used for the 2-class effect size, and the standardized mean difference (SMD) is used for the continuity effect. The 95% confidence interval (CI) is used. The mesh meta-analysis used a consistency model with a statistical significance of *P* < .05. The inconsistency test uses a node analysis model (i.e., the point method). If *P* > .05, there is no evidence to prove that there is inconsistency in the study. The convergence of the mesh Meta is tested by the potential scale reduction parameter (PSRF). If the PSRF is close to 1, it means that the convergence of this study is good, and the conclusion of the meta-analysis is reliable.

#### 2.2.10 Quality of evidence

This study will use the GRADE pro online software to evaluate the included studies.

## 3 Discussion

The cause of PE has not been fully clarified.^[26,27]^ It is currently believed that the disease may have the following pathogenic factors: psychological reasons: self-confidence, uneasiness, loss of self-confidence in sexual intercourse. Organic causes: due to penile hypersensitivity or increased sensory nerve excitability, the ejaculation center is caused by the dysfunctional function of the penis. Others: such as dermatitis, balanitis, prostatitis, seminal vesiculitis, urethritis and other inflammatory diseases, sympathetic ganglia damage, polycythemia, drug withdrawal syndrome. Due to the uncertainty of its etiology, treatment methods are also diversified. For example, sexy concentration training is the most representative program in psychotherapy, and a satisfactory success rate has been achieved. However, due to the long-term close cooperation of the woman. The study found that local anesthetics (such as lidocaine-propylaminocaine cream) can reduce the sensitivity of the glans and prolong the IELT, but its spread can cause numbness to the vaginal wall of the spouse, which limits its wide application. Some surgical methods, such as penile prosthesis implantation and penile dorsal nerve resection, have begun trials in some countries, but their safety and effectiveness have yet to be further studied. Selective 5-HT reuptake inhibitors (SSRIs) were originally drugs for the treatment of depression, with varying degrees of delayed ejaculation, have been shown to have a definite therapeutic effect on the disease, and have been identified as the drug of choice for the treatment of this disease.^[28]^ Such drugs include citalopram, fluoxetine, fluvoxamine, paroxetine, and sertraline, which have similar drug mechanisms, can extend IELT to varying degrees, and improve the sexual life satisfaction of patients and spouses. Among them, paroxetine is the most effective, and some studies have found that after treatment, the patient's IELT can be extended by 8 times.^[29]^ However, these drugs generally have many adverse reactions, such as fatigue, yawning, nausea, vomiting, dry mouth, and so on. As an external treatment with low side effects and environmental protection, acupuncture has long been used to treat male diseases such as erectile dysfunction and chronic prostatitis. Although acupuncture treatment of premature ejaculation lacks understanding, clinical studies have shown that acupuncture can prolong ejaculation to a certain extent. At present, the clinical evidence of acupuncture for premature ejaculation is insufficient. Most of them are the result of low quality, small samples. Therefore, we will use systematic reviews and meta-analyses to assess the efficacy and safety of acupuncture for premature ejaculation. The study also has the following defects: the minimum sample content is not estimated in the included studies, the sample size is small, and false positive or false negative results may occur; the age level is not refined in the study, the duration of the disease is different, and the intervention measures (drugs), different choices, treatment time, etc., will have a certain impact on the evaluation results, which will affect the results and the strength of their argumentation.

## Author contributions

**Data curation:** Hengheng Dai, Jisheng Wang.

**Formal analysis:** Binghao Bao, Yubing Yan.

**Funding acquisition:** Haisong Li.

**Project administration:** Bin Wang.

**Supervision:** Song Sun, Bin Wang.

**Validation:** Haisong Li.

**Writing – original draft:** Hengheng Dai, Jisheng Wang.

**Writing – review & editing:** Binghao Bao, Song Sun.
